# Antcin-H Isolated from* Antrodia cinnamomea* Inhibits Renal Cancer Cell Invasion Partly through Inactivation of FAK-ERK-C/EBP-*β*/c-Fos-MMP-7 Pathways

**DOI:** 10.1155/2017/5052870

**Published:** 2017-11-02

**Authors:** Kun-Yuan Chiu, Tzu-Hsiu Chen, Chi-Luan Wen, Jin-Mei Lai, Chi-Chih Cheng, Hsiang-Chun Liu, Shih-Lan Hsu, Yew-Min Tzeng

**Affiliations:** ^1^Institute of Biochemical Sciences and Technology, Chaoyang University of Technology, Taichung, Taiwan; ^2^Division of Urology, Department of Surgery, Taichung Veterans General Hospital, Taichung, Taiwan; ^3^Department of Applied Chemistry, National Chi Nan University, Puli, Nantou, Taiwan; ^4^Department of Health and Nutrition, Chia Nan University of Pharmacy & Science, Tainan, Taiwan; ^5^Taiwan Seed Improvement and Propagation Station, Council of Agriculture, Propagation Technology Section, Taichung, Taiwan; ^6^Department of Life Science, Fu-Jen Catholic University, New Taipei City, Taiwan; ^7^Department of Medical Research, Taichung Veterans General Hospital, Taichung, Taiwan; ^8^Department of Life Science, National Taitung University, Taitung, Taiwan

## Abstract

Antcin-H, a natural triterpene, is purified from a famous anticancer medicinal mushroom,* Antrodia cinnamomea,* in Taiwan. This study showed that antcin-H inhibited the growth of human renal carcinoma 786-0 cells; the IC_50_ value (for 48 h) was 170 *μ*M. Besides, the migration and invasion of 786-0 cells were suppressed by antcin-H under noncytotoxic concentrations (<100 *μ*M); these events were accompanied by inhibition of FAK and Src kinase activities, decrease of paxillin phosphorylation, impairment of lamellipodium formation, and upregulation of TIMPs and downregulation of MMPs, especially MMP-7 expression. Luciferase reporter assay showed that antcin-H repressed the MMP-7 promoter activity, in parallel to inhibiting c-Fos/AP-1 and C/EBP-*β* transactivation abilities. Moreover, antcin-H suppressed the activity of ERK1/2 and decreased the binding ability of C/EBP-*β* and c-Fos on the upstream/enhancer region of MMP-7 promoter. Overall, this study demonstrated that the anti-invasive effect of antcin-H in human renal carcinoma 786-0 cells might be at least in part by abrogating focal adhesion complex and lamellipodium formation through inhibiting the Src/FAK-paxillin signaling pathways and decreasing MMP-7 expression through suppressing the ERK1/2-AP-1/c-Fos and C/EBP-*β* signaling axis. Our findings provide the evidence that antcin-H may be an active component existing in* A. cinnamomea* with anticancer effect.

## 1. Introduction

Human renal cell carcinoma (RCC), the second common but most lethal cancer of urologic origins, is relatively rare compared to the other carcinomas, but the incidence of RCC is increasing [1]. Although RCC is curable when it is diagnosed in the very early stage of the disease [2], due to its asymptomatic clinical course, by the time of diagnosis about 25% of RCC patients present with invasion of the tumor to the surrounding tissues and distant metastasis [3, 4]. New treatment modalities including immunotherapies with interferon or interleukin-2 and targeting therapies focusing on vascular endothelial growth factor and mTOR pathway have been developed recently for the patients with metastatic diseases [5, 6]. Nevertheless, relatively higher costs and unpredictable side effects limit the clinical uses of all these potential treatment options. Nowadays, no agent can be clinically used to prevent or treat the metastatic RCC and most patients ultimately succumb to metastatic disease [7, 8]. Therefore, an effective therapeutic strategy is a critical issue in the management of these patients.

Cancer metastasis is complex and complicated process that involves several classes of proteins, including adhesion molecules and extracellular proteases. Classical and important metastatic proteins are matrix metalloproteinases (MMPs); numerous reports show that MMPs are overexpressed in metastatic human tumors [9–12]. MMPs can degrade extracellular matrix components and their overexpression correlates with metastasis and poor prognosis in most tumor types [13, 14]. Among the MMPs, MMP-7, known as matrilysin, has a broad spectrum of proteolytic activity capable of cleaving various types of extracellular matrix [15]. Strong correlations between MMP-7 overexpression and invasion are observed in cancer cell lines and mouse models across most tumor types [16, 17]. It has been demonstrated that MMP-7 is preferentially expressed at the invasive front of tumors [11, 18], and its overexpression associates with metastatic disease and unfavorable outcome in RCC [11, 12]. Overall, these findings make MMP-7 a strong and novel target for pharmacological antimetastasis therapy in RCC.

Fungi provide a huge resource and have been used for an effective way to develop new pharmaceutical products. Several studies have shown the potentials of* Antrodia cinnamomea*, a well-known medical mushroom in Taiwan, on prevention and treatment of liver diseases, food and drug intoxication, hypertension, and cancers [19]. Several compounds have been isolated and identified from fruiting bodies of* A. cinnamomea* including benzenoids, steroids, and triterpenoids [19, 20]. The precise compounds and their mode of actions responsible for the observed biological functions have been studied recently [21–24]. Antcins, steroid-like compounds, exert anti-inflammatory effect and enhance blood circulation [25]. A recent report demonstrates that antcin K triggers intrinsic apoptotic cell death through the mitochondrial and endoplasmic reticulum stress-induced signaling pathways [26]. However, there is no study exploring the effects of antcin-H, a pure compound isolated from* A. cinnamomea*, on human cancer cells. This study was aimed to examine the anticancer effect and its molecular mechanism of antcin-H in human RCC cells. Our results firstly showed that antcin-H inhibited the Src/FAK/paxillin and Src/FAK/ERK-c-Fos-C/EBP-*β* signaling pathways to impair lamellipodium formation and decrease MMP-7 expression, consequently suppressing RCC cell migration and invasion, suggesting that antcin-H might have the potential for treating metastatic RCC.

## 2. Materials and Methods

### 2.1. Isolation of Antcin-H

Antcin-H used in this study was provided by Professor Yew-Min Tzeng at Natural Products and Bioprocess Laboratory, the Institute of Biochemical Sciences and Technology, Chaoyang University of Technology, Taiwan. In brief, the natural product antcin-H was isolated from the cultivated fruiting bodies of* A. cinnamomea* YMT 1002 (GenBank KJ704843). The powdered fruiting bodies (30 g) were extracted through a serial solvent extraction and silica gel column chromatography operations; 65 mg yellow needle antcin-H was obtained with yield of 0.21% (W/W). All the 1H and 13C NMR spectral data derived from antcin-H were in complete accord with the assigned structure. The isolation method of the natural product antcin-H was reported by Tzeng's laboratory previously [27]. The purity (>95%) of antcin-H was confirmed by HPLC analysis [28].

### 2.2. Cell Culture and Viability Assay

Renal carcinoma 786-0 cell line was cultured in Roswell Park Memorial Institute 1640 medium (RPMI 1640) supplemented with 10% fetal bovine serum, 2 mM L-glutamine, 1 mM sodium pyruvate, 1% nonessential amino acid, and 1 mM HEPES. Cells were incubated in 95% air, 5% CO_2_ humidified atmosphere at 37°C. Cell viability was performed with trypan blue exclusion method. Briefly, 786-0 cells plated in 12-well plate and treated with various dosages of antcin-H (0, 20, 50, 100, 200, and 300 *μ*M) for indicated times. The viable cells were determined by trypan blue reagent.

### 2.3. Wound-Healing Assay in Live Cells

The* in vitro* wound-healing assay was performed by using IBIDI culture-insert (IBIDI, Martinsried, Germany) to create a defined cell gap. Briefly, without or with antcin-H treatment, 786-0 cells were trypsinized and seeded in the wells of the culture-insert containing 10% serum medium and grown until 95% confluence. For the cell culture, a linear wound was then created and treated without or with antcin-H in a low serum condition; to obtain migrating live cell imaging in wounded region, the Olympus microscope was used with a 40x objective. The image of cell migration into the wound front was microphotographed every 10 min for up to 24 h.

### 2.4. Migration and Invasion Assay

With or without antcin-H treatment, 786-0 cells were trypsinized and loaded in the upper chamber of the Transwell apparatus (pore size: 8 *μ*m; Millipore, Billerica, MA, USA). After treatment, the upper chamber cells were scraped off, the filters were then washed and fixed and stained with Giemsa solution, and then the migrated cells were counted. For invasion assay, cells were loaded onto a matrigel (BD Biosciences, San Jose, CA, USA) precoated Transwell at a density of 2 × 10^4^ cells with serum-free medium, whereas RPMI containing 10% FBS was added to lower chamber as a chemoattractant. After incubation, the cells that invaded across the matrigel to the lower surface of membrane were fixed in methanol and stained with Giemsa solution, and then the invaded cells were measured.

### 2.5. Western Blot Analysis

Cells were harvested by scrapping with iced cold PBS and lysed directly in RIPA buffer containing 50 mM Tris-HCl (pH 7.4), 150 mM NaCl, 1% Triton X-100, 0.25% sodium deoxycholate, 5 mM EDTA (pH 8.0), and 1 mM EGTA and supplemented with protease and phosphatase inhibitors at 4°C for 20 min and then centrifuged with 12000 rpm at 4°C for 30 min and total protein content was determined by Bradford assay. Equal amounts of total proteins were separated by SDS-polyacrylamide gel electrophoresis. Immunoblotting was performed using primary antibodies against Src, phosphorylated-Src (p-Src), paxillin, and phosphorylated-paxillin (p-PXN) (Cell Signaling Technology, Beverly, MA) and ERK1/2, phosphorylated-ERK1/2 (p-ERK1/2), c-Fos, C/EBP-*β*, phosphorylated-C/EBP-*β* (p-C/EBP-*β*), FAK, and phosphorylated-FAK (p-FAK) (Santa Cruz, CA, USA). The image was investigated using an ECL-Plus detection kit (PerkinElmer Life Sciences, Inc., Boston, MA, USA).

### 2.6. Immunofluorescence Assay

Cells were cultured on 15 mm microscope cover glasses in 12-well plate. After antcin-H was exposed for indicated time, the cells were fixed with 4% paraformaldehyde and permeabilized by 0.1% Triton X-100 at room temperature. Samples were blocked with 1% BSA for 30 min and incubated with primary antibodies against phosphorylated-FAK and phosphorylated-paxillin overnight. After being washed with PBS, the cells were incubated with Alexa-Fluor 488-conjugated secondary antibody (Invitrogen, Carlsbad, CA, USA), and then the actin image was investigated using Phalloidin-iFluor 647 Conjugate (AAT Bioquest®, Inc., CA) and, finally, incubated with DAPI (Molecular Probes Inc., Eugene, Oregon, USA) for nucleus detection. The cells were then examined using a laser confocal microscope (FV1000, Olympus).

### 2.7. Quantitative-PCR and RT-PCR

786-0 cells were exposed to antcin-H or control solvent for indicated time and total RNA was extracted by using TRIzol® isolated kit (Invitrogen, Carlsbad, CA, USA) following the manufacturer's instruction. One *μ*g of total RNA was reverse-transcribed using the First-Strand cDNA Synthesis kit (Thermo Fisher Scientific Inc.). The cDNA products of RT-PCR were then operated with ABI PRISM 7900 Sequence Detector System to determine the mRNA levels according to the manufacturer's instructions. The quantitative RT-PCR reaction mixture contains cDNA, primers, and SYBR Green PCR master mix (Applied Biosystems, Life Technologies). *β*-Actin was used as an internal loading control. The used primer sequences were listed as follows: MMP-2: forward 5′-CTTCCAAGTCTGGAGCGATGT-3′, reverse 5′-TACCGTCAAAGGGGTATC CAT-3′; MMP-3: forward 5′-GAGGCATCCACACCCTAGGTT-3′, reverse 5′-TAGC TACGTCGGTAAAGACTA-3′; MMP-7: forward 5′-GGAGGAGATGCTCACTTC GAT-3′, reverse 5′-AGGAATGTCCCATACCCAAAGA; MMP-13: forward 5′-AAG GAGCATGGCGACTTCT-3′, reverse 5′-TGGTTCAGGAAAAGC; tissue inhibitors of metalloproteinases 3 (TIMP-3): forward 5′-CAGGACGCCTTCTGCAA-3′, reverse 5′-CCCCTCCTTTACCAGCTTCTTC-3′; TIMP-4: forward 5′-CACCCTCAGCAGCAC ATCTG-3′, reverse 5′-GGCCGGAACT ACCTTCTCACT-3′.

### 2.8. Chromatin Immunoprecipitation (ChIP) Assay

ChIP assay was conducted as described previously [29]. Briefly, after treatment, cells were collected and fixed with 1% formaldehyde at 37°C for 10 min then treated with glycine to quench the cross-links. Cells were harvested by ice PBS containing proteinase inhibitor and lysed with SDS lysis buffer and then sonicated with the following condition: 20% amplitude for 10 seconds and rest for 10 seconds, repeated 5 times. Lysates were preincubated with the salmon sperm DNA-protein A agarose (Millipore, Billerica, MA, USA) and subjected to immunoprecipitation overnight at 4°C with normal IgG or antibody against C/EBP-*β* (Cell Signaling Technology, Beverly, MA) or c-Fos (Santa Cruz, CA, USA). Precipitates were washed and eluted. The chromatin extracted and protein-DNA cross-links reversed by NaCl. Then DNA was purified by DNA clean-up purification kit (Promega, Madison, WI, USA), and the relative amount of DNA sequence from the MMP-7 promoter region was estimated by PCR analysis. The used primer sequences were listed as follows: for −1.4 kb length, 5′-TGAGCTACAGTGGGAACAGG-3′ and 5′-TCATCGAAGTGAGCATC TCC-3′; for −876 to −1201 bp region, sense: 5′-CTCCAGCATATTTGGAGTGTTTCC-3′ and antisense: 5′-CTTCCAATCA CTCTGACTCTGGC-3′; for −263 to −529 bp region, sense: 5′-CATCTTTCCCCTGTATGGAGAAC-3′ and antisense: 5′-GACTGCTCTCAT AGGTATCATTCAGG-3′; for −138 to −288 bp region, sense: 5′-CCTGAATGATACCTA TGAGAGCAG-3′ and antisense: 5′-CGAGGAAGTATTACATCGTTATTGG-3′; for +2 to −229 bp region, sense: 5′-GGAGTCAATTTATGCAGCAGACAG-3′ and antisense: 5′-GGTGTTTTCTGCTAGT GACTGCAG-3′.

### 2.9. Luciferase Reporter Assay

To generate luciferase reporter, the sequence of MMP-7 containing MluI and BglII restriction site was cloned into the upstream of firefly luciferase gene in PGL3 vector. The primers were +15 to −1000 bp: 5′-ACGCGTTCATTTTTGGTAAGAATGGTCATTGG-3′ (forward) and 5′-AGATCTTATGGTTGATTTGGTGTTTTCTGCTAG-3′ (reverse). After cloning, the vectors were sequenced to confirm the orientation and integrity of the inserts of each construct. For transfection, cells were cotransfected with the vector DNA and the pRL-CMV internal control which contained the Renilla luciferase gene by Lipofectamine 2000 (Invitrogen, Waltham, MA, USA) and then added with or without antrocin into each well. Cells were collected and analyzed for luciferase activity with the Dual-Luciferase Reporter Assay System kit (Promega, Madison, WI, USA).

### 2.10. Statistical Analysis

The results were confirmed by carrying out at least three independent experiments with similar pattern. Values were expressed as means ± SD from three separate experiments (*n* = 9). Statistical comparisons were done using one-way analysis of variance (ANOVA) with Student's* t*-test, the statistical significance was set at ^*∗*^*p* < 0.05, ^*∗∗*^*p* < 0.01, and ^*∗∗∗*^*p* < 0.001.

## 3. Results

### 3.1. Growth Inhibitory Effects of Antcin-H

**Figure 1:**
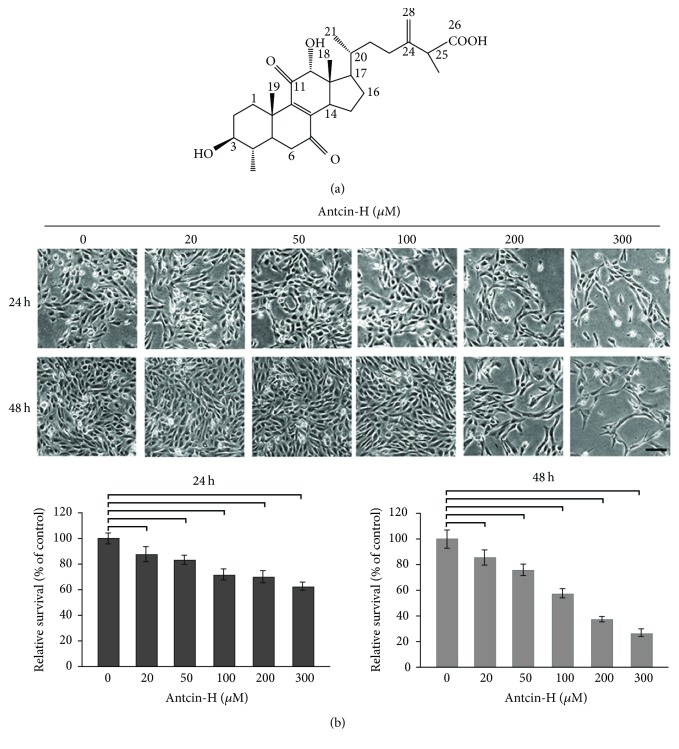
Growth inhibitory effect of antcin-H. (a) The chemical structure of antcin-H. (b) Human RCC 786-0 cells were treated with various concentrations (0, 20, 50, 100, 200, and 300 *μ*M) of antcin-H for 24 and 48 h. After incubation, cell morphology was investigated using phase-contrast microscope (upper panel). Scale bar, 100 *μ*m. The cell viability was determined by trypan blue dye exclusion method (lower panel).

The chemical structure of antcin-H is shown in Figure 1(a). The effects of antcin-H on renal cancer cell proliferation and human renal carcinoma 786-0 cells were treated with antcin-H. As depicted in Figure 1(b), antcin-H inhibited 786-0 cell growth dose-dependently, and the IC_50_ value was 170 *μ*M after 48 h exposure. Incubation of 786-0 cells with lower concentrations of antcin-H (<200 *μ*M) caused a growth inhibitory effect but did not display any signs of cytotoxicity by morphological investigation. However, administration of 300 *μ*M antcin-H resulted in rounding and detaching due to cytotoxicity.

### 3.2. Inhibition of Migration and Regulation of Migration-Related Molecules by Antcin-H

**Figure 2:**
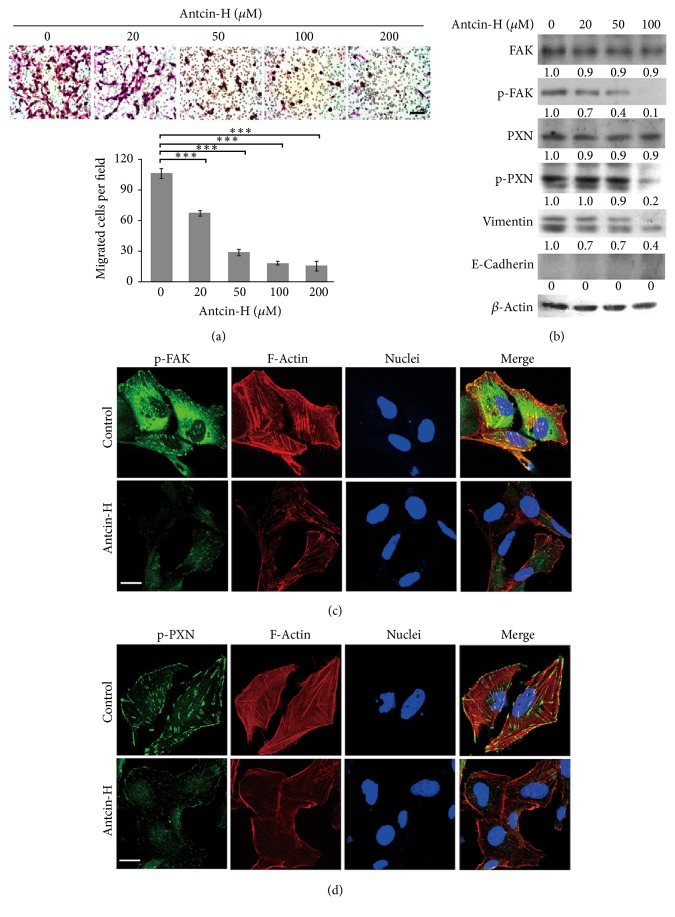
Inhibition of cell migration and modulation of migration-related proteins by antcin-H in vitro. (a) 786-0 cells were treated without or with 20, 50, 100, and 200 *μ*M antcin-H for 24 h, and then cells were seeded in the upper part of Transwell. After 16 h, cells on the bottom side of the filter were microphotographed and counted. Data were represented as the mean ± SD of three independent experiments. Statistically significant, ^*∗∗∗*^*p* < 0.001. Scale bar, 100 *μ*m. (b) Regulation of FAK, paxillin, E-cadherin, and vimentin by antcin-H. 786-0 cells were treated without or with 20, 50, and 100 *μ*M antcin-H for 24 h, and then protein lysates were isolated. The levels of phosphorylated-FAK, phosphorylated-paxillin, E-cadherin, and vimentin were examined by Western blot analysis. *β*-Actin was used as an internal loading control. Confocal imaging of (c) phosphorylated-FAK and (d) phosphorylated-paxillin. 786-0 cells were treated without or with 100 *μ*M antcin-H for 24 h. The cellular distribution of phosphorylated-FAK and phosphorylated-paxillin was examined by immunofluorescence staining using phosphorylated-FAK and phosphorylated-paxillin specific antibodies. The immunoreactive images were investigated by confocal microscope. Scale bar, 20 *μ*m.

Metastasis has been considered as a poor prognostic factor in RCC [30]; therefore, developing safe and effective therapeutic agents for the treatment of metastatic RCC is urgently required. To examine the effect of antcin-H on cell migration, Transwell migration assay was carried out. As shown in Figure 2(a), treatment of 786-0 cells with noncytotoxic concentrations of antcin-H for 24 h retarded cell migration in a concentration-dependent manner; the number of migrated cells was markedly reduced upon antcin-H treatment.

Previous study demonstrates that FAK/paxillin pathway plays a key role in formation of focal adhesion contact and concomitant cell migration and invasion [31]. Besides, epithelial mesenchymal transition (EMT) is an essential process for cancer cell to acquire migration and invasion ability [32]. Loss of E-cadherin and increase of vimentin are surrogate markers for cell migration and invasion in EMT process. To explore the molecular mechanisms involved in the inhibitory effect of antcin-H on cell migration, the expressed levels of FAK, paxillin, E-cadherin, and vimentin were examined by Western blot analysis. As depicted in Figure 2(b), antcin-H induced a dose-dependent decrease in phosphorylated FAK^Tyr925^ and paxillin^Tyr118^ levels, with a significant change observed at 50 and 100 *μ*M, which is consistent with doses that are antimigration (Figure 2(a)). Moreover, the expressed level of vimentin was significantly reduced by antcin-H. However, the levels of total FAK and paxillin were not affected by antcin-H. Moreover, the expression of E-cadherin could not be detected in both control and antcin-H-treated 786-0 cells (Figure 2(b)). These results reveal that FAK, paxillin, and vimentin may be targeting molecules involved in antcin-H-mediated inhibition of 786-0 cell migration.

To further examine the cellular distribution of phosphorylated-FAK and phosphorylated-paxillin, 786-0 cells were treated with 100 *μ*M antcin-H and subjected to immunostaining analysis with anti-phosphor-FAK and anti-phosphor-paxillin antibody and then counterstained with phalloidin-rhodamine and DAPI for actin and nucleus staining, respectively. Confocal imaging revealed that the majority of control untreated cells showed small dot-like structures of phosphorylated-FAK (Figure 2(c)) and phosphorylated-paxillin (Figure 2(d)) extending into a polarized, actin-containing lamellipodia. In contrast, antcin-H treatment decreased the phosphorylated-FAK and phosphorylated-paxillin levels at the leading edge and reduced lamellipodia formation in 786-0 cells (Figures 2(c) and 2(d)).

**Figure 3:**
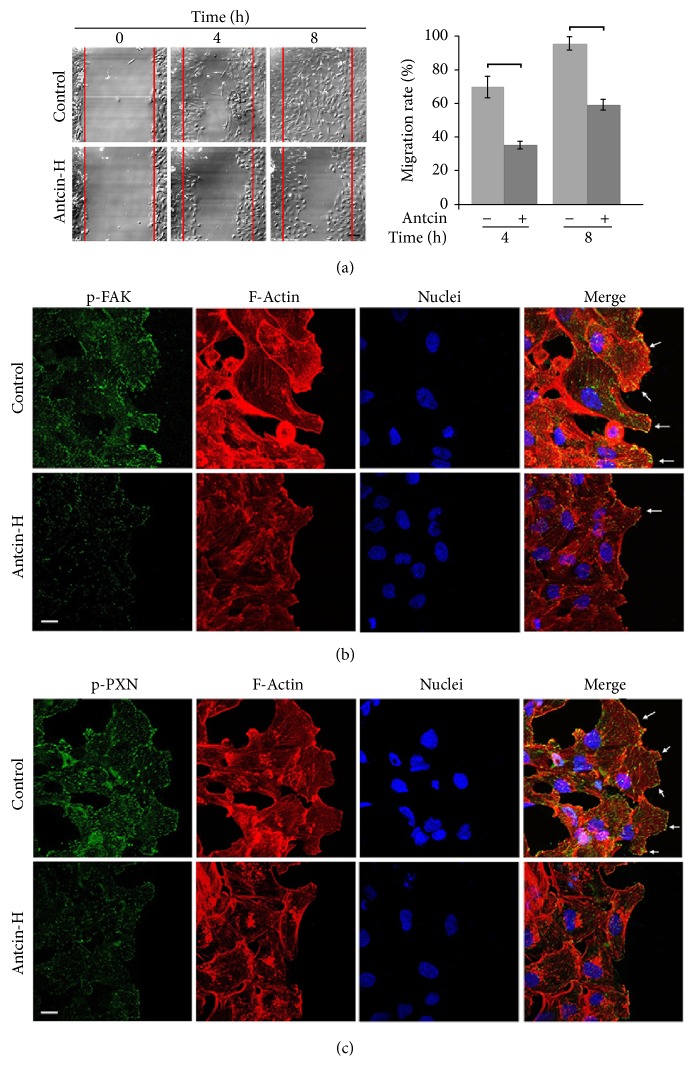
Suppression of wound-healing and disruption of lamellipodium formation by antcin-H. (a) Live cell time-lapse images at wound-healing front. 786-0 cells were cultured to 100% confluence on glass coverslips. After wound was made, fresh media without or with 100 *μ*M antcin-H were added. Cells were allowed to migrate; the time-lapse of live cell imaging was observed at 4 and 8 h. Scale bar, 20 *μ*m. (b) Immunostaining with FAK or (c) paxillin antibody. After 8 h wounding, the migrated cells were fixed, and the immunofluorescence staining was carried out using anti-phosphorylated-FAK and anti-phosphorylated-paxillin antibodies. The immunoreactive image was recorded by confocal microscope. Arrow, formation of lamellipodium. Scale bar, 20 *μ*m.

Next, the antimigratory activity of antcin-H was evaluated by real-time, live cell imaging of wound-healing assay. 786-0 cells were incubated with 100 *μ*M antcin-H for indicated time periods. In representative time-lapse images, antcin-H treatment significantly retarded cell migratory activity (Figure 3(a)). Because both FAK and paxillin are the important regulators of lamellipodial dynamics in motile cells [33], the formation of lamellipodium at the wound margin was investigated by immunostain with anti-phosphorylated-FAK and phosphorylated-paxillin antibody. Small dot-like structures, regarded as FAK (Figure 3(b)) and paxillin (Figure 3(c)) localized at the front edge, which associated with lamellipodium containing bundles of F-actin were detected in untreated 786-0 cells. Conversely, only a few small dot-like FAK and paxillin immunoreactivity located at front edge of wound margin, and a clear lamellipodia could not be observed in antcin-H-treated 786-0 cells. Since lamellipodia are shown to increase in highly motile cells, these results indicate that antcin-H exposure may cause a decrease of cell migratory capability in 786-0 cells.

### 3.3. Antcin-H Suppresses Cell Invasion and Modulates MMPs and TIMPs Expression

**Figure 4:**
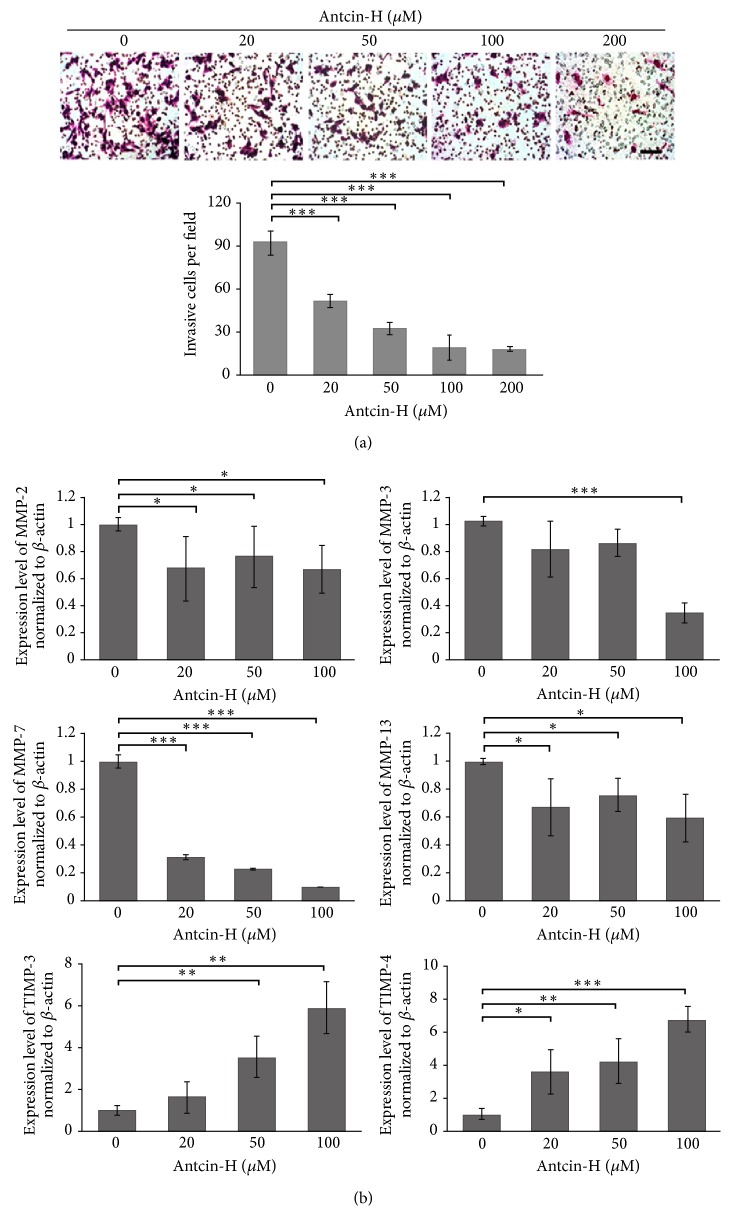
ANTCIN-H prevents invasion and regulates MMPs gene expression. (a) 768-0 cells were pretreated without or with 20, 50, 100, and 200 *μ*M antcin-H for 24 h and then seeded into Matrigel-coated Transwell apparatus for another 24 h in the absence or presence of antcin-H. After incubation, the invaded cells were stained with Giemsa solution and counted using a microscope. Scale bar, 100 *μ*m. The data were represented as the mean ± SD of nine replicates from three separated experiments. ^*∗∗∗*^*p* < 0.001 versus control. (b) Real-time PCR analysis of MMPs and TIMPs gene expression. Cells were treated without or with 20, 50, and 100 *μ*M antcin-H for 24 h. After treatment, the RNA extracted from 786-0 cells was subjected to a real-time PCR. *β*-Actin was used as an internal control. Data were represented as the mean ± SD of three independent experiments. Statistically significant, ^*∗*^*p* < 0.05, ^*∗∗*^*p* < 0.01, and ^*∗∗∗*^*p* < 0.001.

To examine whether antcin-H could suppress cell invasion in RCC 786-0 cells, Matrigel-coated Transwell invasion assay was conducted, and invaded cells were photographed using a microscope with a 20x objective lens. Representative images of invaded cells were shown in Figure 4(a), antcin-H effectively impaired the invasive capability of 786-0 cells, and the number of invaded cells was significantly fewer than those in untreated control. At 100 *μ*M antcin-H treatment revealed an approximately 80% decreased invasive ability in comparison with untreated control. Because the expression of MMP and TIMP family members are critical for cancer invasion, to characterize the regulation of* MMP*s and* TIMP*s genes by antcin-H, real-time PCR analysis was carried out. Results indicated that* MMP-2*,* MMP-3*,* MMP-7*, and* MMP-13* genes were downregulated, whereas* TIMP-3* and* TIMP-4* were upregulated after exposure to antcin-H (Figure 4(b)). However,* MMP-1*,* MMP-8*,* MMP-9*,* MMP-10*,* MMP-11*,* TIMP-1,* and* TIMP-2* genes were not altered by antcin-H (data not shown).

### 3.4. Antcin-H Inhibits the ERK Signaling Pathway

**Figure 5:**
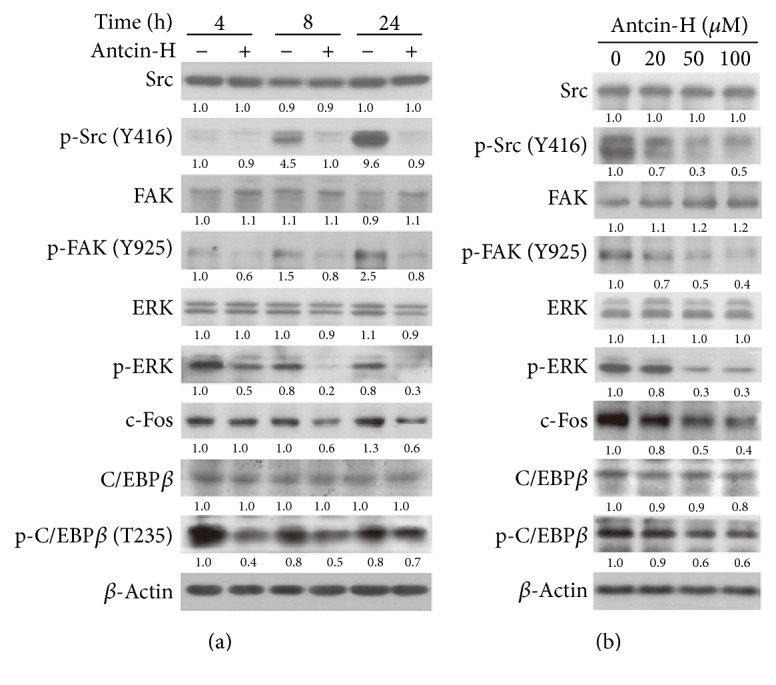
Suppression of Src, FAK, and ERK1/2 signaling pathways by antcin-H. (a) Time course-dependent experiment. 786-0 cells were treated without or with 100 *μ*M antcin-H for 4, 8, and 24 h. (b) Dose-dependent experiment. Cells were treated without or with 20, 50, and 100 *μ*M antcin-H for 24 h. After incubation, total protein lysates were isolated; the Western blotting analysis was performed to examine the levels of phosphorylated-Src, phosphorylated-FAK, phosphorylated-ERK1/2, phosphorylated-C/EBP-*β*, and c-Fos. *β*-Actin was used as an internal loading control.

To further characterize the possible mechanism that underlies the inhibitory effect of antcin-H on 786-0 cells, the activation of several kinases involving FAK pathway and contributing to cell migration and invasion, including Src, FAK, and ERK1/2, was examined by Western blot analysis. As shown in Figure 5(a), the phosphorylated forms of major FAK signaling pathways, including FAK, Src, and ERK1/2, were drastically decreased after exposure to 100 *μ*M antcin-H for 4 h and continuously suppressed at 24 h. Consistently, the major ERK downstream transcription factors, such as c-Fos and phosphorylated-C/EBP-*β*, were also time- and dose-dependently reduced in response to antcin-H treatment (Figures 5(a) and 5(b)). However, the amount of total FAK, Src, and ERK1/2 did not change when incubated with antcin-H.

### 3.5. Inhibition of c-Fos and C/EBP-*β* DNA Binding Activity and Transactivation Ability Contributes to Antcin-H-Mediated MMP-7 Downregulation

**Figure 6:**
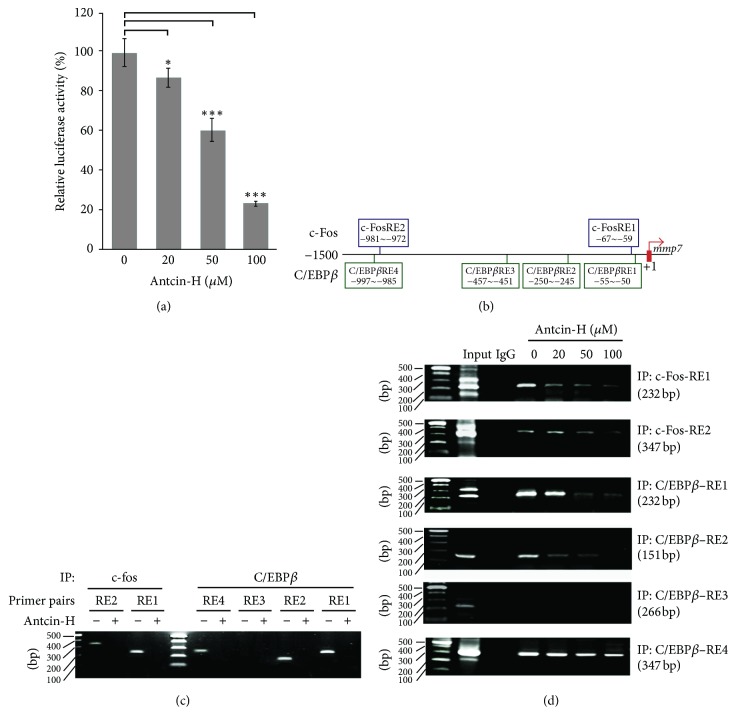
Inhibition of* c-Fos *and* C/EBP-β* activities involved in antcin-H-mediated* MMP-7* downregulation. (a) Reporter luciferase assay. 786-0 cells were transiently transfected with reporter vector containing* MMP-7* promoter +1~−1500 region or control vector for 24 h, and then the cells were treated without or with 20, 50, and 100 *μ*M antcin-H for another 24 h. After incubation, the luciferase activity was measured and the relative luciferase activity was presented as means ± SD. Statistically significant, ^*∗*^*p* < 0.05, ^*∗∗*^*p* < 0.01, and ^*∗∗∗*^*p* < 0.001. (b) Putative binding sites of c-Fos and C/EBP-*β* located at upstream of* MMP*-7 promoter. (c) Antcin-H prevents c-Fos and C/EBP-*β* binding to the* MMP-7* upstream promoter/response element region. The 786-0 cells were treated without or with 100 *μ*M antcin-H for 24 h, the cells were collected, and then ChIP assay was carried out. (d) Antcin-H dose-dependently inhibits c-Fos and C/EBP-*β* binding to the* MMP-7* upstream promoter/response element region. The 786-0 cells were treated without or with 20, 50, and 100 *μ*M antcin-H for 24 h, the cells were collected, and then the activity of c-Fos and C/EBP-*β* binding to each response site located at* MMP-7* promoter upstream was determined by ChIP assay.

Growing evidence indicates that MMP-7 is overexpressed in RCC [11] and was clinically associated with metastasis and poor prognosis in patients with RCC [11, 12]. The present study showed that the expression of* MMP-7* gene was unusually reduced following antcin-H administration (Figure 4(b)); to further confirm that the antcin-H-mediated MMP-7 downregulation is regulated at transcriptional level, the promoter activity of MMP-7 was evaluated by the reporter luciferase assay. As depicted in Figure 6(a), antcin-H suppressed reporter activity in 786-0 cells in a concentration-dependent manner. These data suggest that antcin-H reduced* MMP-7* gene expression through repression of* MMP-7* promoter activity.

Notably, there are two c-Fos/AP1 (c-FosRE1, −67~−59 and c-FosRE2, −981~−972) and four C/EBP*β* (C/EBP-*β*RE1, −55~−50; C/EBP-*β*RE2, −250~−245; C/EBP-*β*RE3, −457~−451; C/EBP-*β*RE4, −997~−985) putative binding sites locate upstream of* MMP-7* promoter (Figure 6(b)). To determine which binding site associated with antcin-H-mediated inhibition of* MMP-7* gene expression, 786-0 cells were incubated without or with 100 *μ*M antcin-H for 16 h, and then anti-c-Fos or anti-C/EBP-*β* antibody was used to carry out chromatin immunoprecipitation (ChIP) assay. As shown in Figure 6(c), in untreated 786-0 cells, c-Fos appeared to be binding on two potential c-Fos/AP1 response sites, c-FosRE1 and c-FosRE2, located upstream of* MMP-7* promoter. The interaction of c-Fos to c-FosRE1 was stronger than that to c-FosRE2. However, antcin-H treatment resulted in a great decrease in c-Fos binding on both sites. Besides, C/EBP-*β* appeared to bind only three putative response regions (C/EBP-*β*RE1, C/EBP-*β*RE2, and C/EBP-*β*RE4) in untreated cells, whereas exposure to antcin-H significantly diminished the binding of C/EBP*β* on these sites. However, the interaction of C/EBP-*β* with C/EBP-*β*RE3 could not be observed in untreated control cells. Moreover, antcin-H dose-dependently blocked c-Fos and C/EBP-*β* binding to their response DNA sequences located at distal and proximal* MMP-7* promoter region (c-FosRE1, c-FosRE2, C/EBP-*β*RE1, C/EBP-*β*RE2, and C/EBP-*β*RE4), respectively (Figure 6(d)). Additionally, no PCR amplified product was seen in sample which was processed by IgG isotype control-mediated precipitation. These results revealed that antcin-H could decrease the recruitment of both c-Fos and C/EBP-*β* transcriptional factors into the upstream response elements of* MMP-7* promoter, ultimately leading to inhibiting the expression of* MMP-7* gene in 786-0 RCC cells.

## 4. Discussion

RCC is an epithelial malignancy of human kidney; surgery is the major strategy for treating patients with RCC. Unfortunately, approximately 30% of patients with RCC will be diagnosed with metastatic disease. Although target therapy and immunotherapy for treatment of patients with metastatic RCC have shown some positive results, continuous treatments with these drugs are associated with a high incidence of toxic effects and resistance [7], and five-year survival of patients with metastatic RCC is only 10% [34]. Therefore, developing strategies for therapeutic interventions in metastatic RCC is of utmost importance. Numerous natural substances have been found to inhibit progression and metastasis of various types of cancer cell lines and reveal that they might be useful for the treatment of metastatic cancer [35, 36]. The present study showed for the first time that antcin-H, a steroid-like compound isolated from a famous anticancer medicinal mushroom* A. cinnamomea*, inhibited Src, FAK, and ERK1/2 signaling pathways and thereby decreased phosphorylated-paxillin, phosphorylated-C/EBP-*β*, and total c-Fos levels and downregulated vimentin and MMPs expression, finally leading to impaired lamellipodium formation and cellular migration/invasion at nontoxic concentrations in human RCC 786-0 cells.

The FAK-Src complex is a pivotal component of focal adhesion contact, as a critical signaling module controls cell motility and potentiates tumor metastasis [31, 37]. Paxillin localizes at focal adhesion contact and acts as a scaffold molecule providing a platform for FAK and Src, which are involved in cell migration events associated with tumor metastasis [38]. Activated FAK/Src complex phosphorylates cytoskeletal adaptor paxillin which promotes cell migration [39]. FAK-Src expression and function have been associated with the majority of invasive and metastatic human cancers, with poor survival [40, 41]. Therefore, inhibition of FAK-Src function and expression is considered as a potential strategy for cancer therapy [42]. A synthetic small molecule with anticancer effect inhibits FAK, Src, and paxillin expression and activation, leading to the suppression of cell migration in colon cancer cells [43]. Src inhibitors reduce the migration of several types of human cancer cell through blocking Src, FAK, p130CAS, and paxillin activation [44, 45]. Inhibiting FAK/paxillin signaling by a sialyltransferase inhibitor effectively retards cancer cell migration [46]. In agreement with these earlier reports, this study showed that antcin-H significantly impaired cell migration by suppression of the FAK, Src, and paxillin signaling pathways. Besides, FAK, paxillin, and actin filaments play the important role in lamellipodium formation [33]. Since lamellipodium is a cell protrusion which is critical for directional migration in vary types of cell [47]. Here, we showed that antcin-H-mediated inhibition of FAK-Src-paxillin signaling axis and RCC cell migration was accompanied by decrease of lamellipodium, indicating that disruption of the lamellipodium formation as well as inhibition of migration and wound closure attributed to reducing formation of focal adhesion and actin bundles through suppressing FAK-Src-paxillin signaling pathway in antcin-H-treated cells.

Growing evidence demonstrates that FAK/ERK-stimulated signaling activates EMT [48]. EMT is an essential step for cancer cell to acquire migration and invasion ability; loss of E-cadherin and increase of vimentin are surrogate markers for cell migration and invasion in EMT process [32]. E-cadherin is a normal epithelial cell adhesion molecule and is considered as a cancer metastasis suppressor. Nevertheless, methylation and loss of heterozygosity of E-cadherin gene are a common event in advanced renal cell carcinoma tissues and cell lines, including 786-0 cell line, which can lead to inactivation of E-cadherin transcription and loss of E-cadherin protein expression [49]. This is consistent with our observation that E-cadherin could not be detected in 786-0 cells. On the other hand, evidence supports that vimentin is a crucial cytoskeletal component of motile mesenchymal cells, including epithelium-derived metastatic tumor cells. Downregulation of E-cadherin or overexpression of vimentin is strongly associated with metastasis, resulting in poor prognosis [50–52]. In the current study, downregulation of vimentin by antcin-H suggested that a loss of EMT and inhibition of invasion potential might occur in antcin-H-treated 786-0 cells.

Literatures show that the process of tumor growth, invasion, and metastasis is tightly regulated by MMPs in various types of malignant tumors, including RCC [11, 12]. Among MMPs, matrilysin (MMP-7) is mainly expressed in malignant tumor cells and preferentially localized at the invasive front of tumors suggesting that it may facilitate destruction of surrounding extracellular matrix and basement membrane [11, 18, 53]. Previous studies demonstrate that MMP-7 is overexpressed in RCC, and increased MMP-7 expression significantly correlates with the malignant behavior of RCC, including invasion, distant metastasis, poor prognosis, and reduced patient survival [11, 54]. These findings suggest that MMP-7 might be regarded as important targets for metastatic RCC therapy to prevent tumor progression and improve survival. In the current study, antcin-H slightly decreased MMP-2, MMP-3, and MMP-13 gene expression while it did not alter the expression of MMP-1, MMP-8, MMP-9, MMP-10, and MMP-11 mRNA levels (data not shown). Intriguingly, the expression of MMP-7 gene was drastically downregulated by antcin-H in 786-0 cells, revealing that targeted inhibition of MMP-7 gene expression might contribute to impairment of RCC cell invasion upon antcin-H administration. Previous studies have shown that MMP activities modulate at the levels of transcriptional regulation, enzymatic activation, and TIMPs inhibition [55]. TIMPs are endogenous inhibitors of MMPs, consisting of four members, TIMP-1, TIMP-2, TIMP-3, and TIMP-4, and play crucial roles in several processes, including cell proliferation, invasion and migration, angiogenesis, and apoptosis [56]. Deregulated expression of TIMPs has been implicated in tumor invasion and metastasis [56]. Overexpression of TIMPs has been reported to inhibit invasive and progressive potentials of the tumor cells [57]. Among TIMPs, TIMP-3 is a broad inhibitor against all MMPs [58], while TIMP-4 is MMP2 and MMP9 inhibitor [56, 59]. Our results indicated that upregulation of TIMP-3 and TIMP-4 expression, but not TIMP-1 and TIMP-2, might be another potential mechanism that contributes to inhibiting MMPs function in response to antcin-H treatment. Besides, TIMP family members have been shown to have proangiogenic effect. Overexpression of TIMP-1 is associated with VEGF expression and promoting neovascularization in breast carcinoma rats [60]. TIMP-2 inhibits angiogenesis by directly binding to *α*3*β*1 integrin [61, 62] or insulin-like growth factor-1 receptor [63]. TIMP-4 is previously reported as a positive regulator of angiogenesis [64, 65]. Unlike TIMP-1 and TIMP-4, TIMP-3 inhibits angiogenesis by blocking the binding of VEGF to VEGF receptor-2 [66] and suppressing angiotensin II receptor activity [67]. These results suggest the important functions of TIMPs in cancer metastasis and angiogenesis. However, the roles of TIMP-3 and TIMP-4 induced by antcin-H in RCC progression are still poorly understood. Further* in vitro* and* in vivo* studies are needed to examine the effects and molecular mechanisms of antcin-H on TIMP-3/4 regulation and angiogenesis in metastatic RCC.

Previous reports demonstrate that FAK/ERK signaling is not only critical for cell migration and invasion, but is also involved in the regulation of MMPs activity and gene expression [68, 69]. ERK and its downstream transcriptional factor AP-1 play an important role in the regulation of MMPs gene expression [70, 71]. AP-1 is a common transcriptional activator composed of the Jun and Fos family members [72]. Besides, ERK can phosphorylate Elk-1 which subsequently upregulates c-Fos gene expression [73]. Literatures have shown that the MMP-7 expression is controlled by modulating the activation of AP-1 transcription factors, c-Fos and c-Jun, through ERK1/2 signaling pathway [17, 74–76]. In this study, we found that two putative binding sites of c-Fos were located at the promoter region of* MMP-7* (Figure 6(b)). In agreement with these previous studies, our observations provided evidence that antcin-H-inhibited MMP-7 gene expression might be through suppressing ERK/c-Fos signaling axis in RCC 786-0 cells. In addition to AP-1/c-Fos, our results indicated that antcin-H-mediated MMP-7 gene downregulation was also via C/EBP-*β* transcriptional repression. Previous studies demonstrate that phosphorylated-C/EBP-*β* by ERK can activate its transcriptional activity [77]. Our results demonstrated that antcin-H inhibited MMP-7 expression might be also via prevention of ERK-C/EBP-*β* activation. On the basis of these results, we suggest that the anti-invasive activity of antcin-H is in part due to the inhibition of MMP-7 expression, which plays a critical role in cancer invasion and metastasis, through the suppression of ERK-mediated AP-1/c-Fos and C/EBP-*β* activities in RCC cells.

In fact, our results demonstrated that, except MMP-7, antcin-H also inhibited other MMP gene expressions, such as MMP-2, MMP-3, and MMP-13, which play critical roles in cancer cell invasion. Similar to MMP-7, these MMPs were also regulated predominantly at the transcriptional level. On the basis of the composition of* cis*-regulatory elements in their promoters, MMP-2, MMP-3, and MMP-13, also contain AP-1 and C/EBP-*β* binding sites proximal and distal to the transcriptional start site, and their expressions have been shown to be regulated at the transcriptional levels via AP-1 and C/EBP-*β* [78–82]. It is possible that these MMPs are regulated similarly upon antcin-H treatment. Besides, on the basis of present results, it is still unclear how TIMP-3 and TIMP-4 expression is upregulated in response to antcin-H treatment. Several transcription factors binding sites, for example, p53, STATs, PPARs, AP-1/c-Fos, C/EBP-*α*, and C/EBP-*β*, are found in the promoter region of TIMP-3 and TIMP-4. However, which transcription factor contributes to the upregulation of TIMP-3 and TIMP-4 by antcin-H needs to be further examined.

## 5. Conclusion

**Figure 7:**
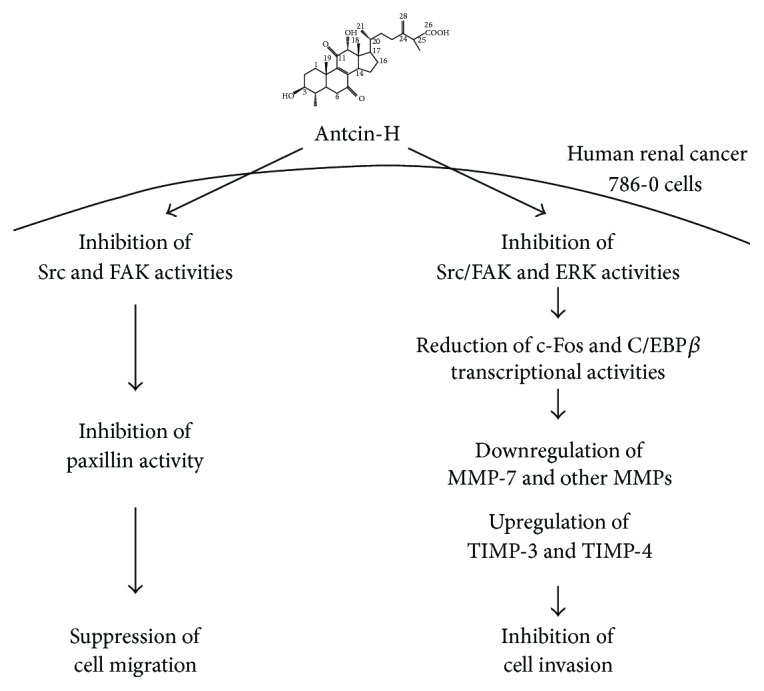
Schematic model of the proposed signaling pathways involved in suppressing cell migration and invasion by antcin-H in human RCC 786-0 cells. Antcin-H inhibits Src, FAK, and ERK1/2 phosphorylated activation, in turn decreasing paxillin, c-Fos, and C/EBP-*β* activities, reducing the binding of c-Fos and phosphorylated-C/EBP-*β* to AP-1 and C/EBP-*β* response elements, thereby decreasing MMPs gene expression, especially MMP-7. Downregulation of MMP-7 and upregulation of TIMP-3 and TIMP-4 gene expression block the degradation of the extracellular matrix proteins and impair the cell invasion. Besides, reducing paxillin phosphorylation and vimentin expression prevents 786-0 cell motility.

Based on our observation, we propose a potential mechanism in antcin-H-treated RCC cells, which shows that antcin-H suppressed FAK-related signaling pathway (Src, FAK, paxillin, and ERK1/2), which impaired focal adhesion turnover and lamellipodium formation, inactivated c-Fos and C/EBP-*β*, downregulated MMPs (especially MMP-7), and upregulated TIMPs (TIMP-3 and TIMP-4) expression (Figure 7). MMP-7 has been considered as a metastatic marker and survival predictor in RCC patients; inhibition of MMP-7 expression and function in tumor cells could be one of the most powerful strategies in metastatic RCC therapy. Our findings provide new insights into the antimigratory and anti-invasive properties of antcin-H and implicate that antcin-H might be a promising phytochemical existing in* A. cinnamomea* with antimetastatic capability in treating advanced RCC.
